# Prevalence and Risk Factors of Renal Stones Among the Bisha Population, Saudi Arabia

**DOI:** 10.7759/cureus.40090

**Published:** 2023-06-07

**Authors:** Akram Bokhari, Ali Amer M Alghamdi, Abdullah Mohammed A Khushayl, Saeed Nasser A Alaklabi, Sarah Khalid A Albarrak, Hadi Abdulaziz Aldarwish

**Affiliations:** 1 Department of Surgery, University of Hail College of Medicine, Hail, SAU; 2 Department of Urology, Miami Cancer institute, Florida, USA; 3 Department of Surgery, University of Bisha College of Medicine, Bisha, SAU

**Keywords:** saudi arabia, nephrolithiasis, calculi, urolithiasis, s: renal stones

## Abstract

Background: In urolithiasis, urinary calculi are formed in the urinary system. Stone development does not initially result in any symptoms, but later renal colic, flank pain, hematuria, obstruction of urine flow, and/or hydronephrosis may indicate renal stone disease. In addition to age, gender, ethnicity, and local climate, urolithiasis can be caused by several other factors. The prevalence and recurrence rate of kidney stone disease is rising globally, while few effective treatment options currently exist.

Methods: Between June and October 2022, a cross-sectional study was conducted. An electronic questionnaire subdivided into three categories was used to determine the prevalence and identify the factors that increase the likelihood of developing urolithiasis among the population in Bisha. The collected data were reviewed and analyzed via IBM Corp. Released 2012. IBM SPSS Statistics for Windows, Version 21.0. Armonk, NY: IBM Corp.

Results: A total of 1,002 participants filled out the questionnaire. The age of the participants ranged from 18 to over 60 years, with an average age of 26.1 ± 13.9 years. There were 451 female participants (45%), and 927 (92.5%) were Saudis. According to the participants' body mass index, 98 (9.8%) were underweight, 388 (38.7%) were normal weight, 300 (29.9%) were overweight, and 216 (21.6%) were obese. The total number of participants with urolithiasis was 161 (16.1%), and 420 (41.9%) had a family history of renal stones. Urolithiasis was found to be significantly associated with family history, smoking, diabetes, hypertension, hyperthyroidism, gout, and chronic kidney disease. Older age and female gender were also associated with the risk of having urolithiasis.

Conclusion: This study found urolithiasis to be highly prevalent among the Bisha population. In terms of risk factors, body mass index, smoking, and diabetes were the most significant. Based on the findings of this study, the authors recommend more public education regarding urolithiasis and its risk factors, emphasizing the importance of preventing the disease and the ways of treating urolithiasis through medical campaigns and social media.

## Introduction

Urolithiasis refers to the development of calculi or stones in the urinary system, which is mainly lodged in the urinary tract. Both the prevalence and recurrence rates of urolithiasis disease are rising globally. Moreover, few effective treatment options exist. Around 12% of people worldwide experience urolithiasis at some point in their lifetime [1-3]. The location of urolithiasis, i.e., whether it occurs in the kidney, ureter, or bladder, determines the pattern of symptoms.

Stone development does not create symptoms at first. However, when developed, renal colic (severe cramping pain), flank pain (pain in the back), hematuria (bloody urine), obstructive uropathy (urinary tract illness), urinary tract infections, obstruction of urine flow, and hydronephrosis become signs and symptoms of stone disease (dilation of the kidney). These ailments may cause nausea, vomiting, and other symptoms related to the stone incident [3]. 

Multiple risk factors may contribute to the development of urolithiasis, such as age, gender, ethnicity, local climate, eating habits, physical activity, and occupation. Comorbidities such as diabetes, high blood pressure, and obesity are other major factors [4,5]. Current research seeks to estimate the epidemiology of kidney stones worldwide. Urolithiasis is a considerable percentage among residents in Saudi Arabia, as an epidemiological study conducted in Saudi Arabia found that among 580 respondents, there is a prevalence of urolithiasis of 9.1% [6].

The pathophysiology of urolithiasis starts with crystal formation in supersaturated urine, which subsequently adheres to the urothelium. Furthermore, urothelial repair takes place after urothelial injury, which increases the chance of further crystal stone formation [7,8]. Most urolithiasis existing in the renal system is composed of calcium salts, mainly calcium oxalate or calcium phosphate. Moreover, uric acid, cystine, and magnesium ammonium phosphate also take place in the renal system [9].

This preventable disease affects 7%-13% of people in North America, 5%-9% of people in Europe, and 1%-5% of people in Asia [6,10]. According to the European Association of Urology, the prevalence of calcium-containing calculi is higher in men, with a male-to-female ratio of 2.7:1 [2,11]. In 2001, an epidemiological study conducted in Riyadh, Saudi Arabia, revealed that Saudis are 2.5 times more likely to develop renal calculi compared to non-Saudis working in the Kingdom [2,12].

In terms of age at diagnosis, the average age in Saudi Arabia is between 22 and 44 years old [6,13]. Once they are formed, they recur within five years. The recurrence among Saudis is as high as 50% [6,14]. Since there is a high prevalence and recurrence rate of preventable urolithiasis worldwide, along with limited studies in the area, this research aims to evaluate the prevalence and risk factors of urolithiasis among the population in Bisha City, Asir, Saudi Arabia.

## Materials and methods

Research design and setting 

The study design is cross-sectional. which was conducted between June and October 2022. The study was designed to assess the prevalence of urolithiasis using an electronic questionnaire written in Arabic and distributed via various social media platforms, primarily Twitter and WhatsApp. Under Google’s privacy policy, information was kept private. The study was carried out in conformity with the Helsinki Declaration’s policy for human-participant studies. The study was approved by the Hail University Research Ethics Committee (H-2022-164).

Sample size 

The formula used to calculate the sample size was ss = (Z2 × p × q)/d2, where ss represents the sample size, Z is 1.96, p is 0.5, q is =1 - p = 0.5, and d is the sampling error at 4%. Using this formula, a minimum sample size of 600 was determined to achieve a study with a 4% error rate and 95% confidence interval (CI). To ensure a greater degree of accuracy, we incorporated a safety margin and ultimately increased the sample size to 1,002, so it was almost double as the safety margin is 10% more.

The inclusion criteria were an age of 18 years or above, living in Bisha, and being willing to participate in the research. Any participants living outside Bisha or younger than 18 years old, along with incomplete submissions, were excluded.

Development and application of the questionnaire

The purpose of the survey was to evaluate the occurrence rate and potential risk factors associated with urolithiasis. The questionnaire consisted of 16 questions, divided into three categories. The first category, comprising eight questions, gathered demographic information about the participants. The second category, consisting of four questions, inquired about the participants' past medical histories. Finally, the third category assesses the self-reported prevalence of urolithiasis by asking them about any history or presenting diagnosis of urolithiasis. 

Language validation was done by a translator translating the survey from English to Arabic. The Cronbach alpha was used to determine the survey's reliability, and it provided a result of reliability with >0.60. The questionnaire was previously used in two different research and was validated [1,2]. Before completing the questionnaire, participants' agreement was obtained.

Data collection

The data were collected from the Bisha population through an electronic questionnaire written in Arabic and distributed via multiple social media applications (mainly Twitter and WhatsApp). According to Google's privacy policy, information is kept private. The study was carried out in conformity with the Helsinki Declaration's policy for human-participant studies, and participant consent was secured before filling out the questionnaire.

Statistical analysis

The collected data was reviewed and subsequently entered into IBM Corp. Released 2012. IBM SPSS Statistics for Windows, Version 21.0. Armonk, NY: IBM Corp. All statistical analyses used the Chi-square test. Every statistical analysis conducted was two-tailed, and a significance level of 0.05 was established, with a p-value less than or equal to 0.05 considered statistically significant.

BMI threshold values specified by the World Health Organization were utilized for bodyweight classification. For study variables such as individuals’ bio-demographic data and medical history items, descriptive analysis was performed by prescribing frequency distributions and percentages. The mean score and percentage score with standard deviation were calculated for quantitative variables. For qualitative variables, a Chi-squared test was used.

Additionally, a multiple regression analysis was used to examine the relationship between various risk factors and the development of stone illnesses. A significance level of 0.05 and a 95% confidence interval (CI) were applied, with a p-value of less than or equal to 0.05 considered statistically significant.

## Results

A total of 1,002 participants completed the questionnaire, with ages ranging from 18 and older and a mean age of 26.1 ± 13.9 years. Females constitute 451 (45%) of the participants, and 927 (92.5%) were Saudi. Five hundred and nine (50.8%) were married, and 683 (68.2%) were university graduates. Regarding body mass index (BMI), 98 (9.8%) were underweight, 388 (38.7%) were normal weight, 300 (29.9%) were overweight, and 216 (21.6%) were obese. Eight hundred and eighteen (81.6%) participants were non-healthcare workers (Table 1). 

**Table 1:** Participants’ bio-demographic information, Saudi Arabia

Bio-demographic data		No	%
Age in years	18-25	310	30.9%
	26-35	262	26.1%
	36-50	311	31.0%
	51+	119	11.9%
Gender	Male	551	55.0%
	Female	451	45.0%
Marital status	Single	435	43.4%
	Married	509	50.8%
	Divorced / widow	58	5.8%
Body mass index	Underweight	98	9.8%
	Normal weight	388	38.7%
	Overweight	300	29.9%
	Obese	216	21.6%
Nationality	Saudi	927	92.5%
	Non-Saudi	75	7.5%
Educational level	Below secondary	24	2.4%
	Secondary	218	21.8%
	University	683	68.2%
	Post-graduate	77	7.7%
Work field	Non-health care worker	818	81.6%
	Health care worker	184	18.4%

Table 2 shows the medical histories of study participants. Two hundred and twenty (22%) were smokers. Eighty-three (8.3%) were hypertensive, 75 (7.5%) had hyperparathyroidism, 69 (6.9%) were diabetic, and 634 (63.3%) had no chronic health problems. One hundred and sixty-one (61.1%) had had renal stones before, and 420 (41.9%) had a family history of renal stones. 

**Table 2:** Medical history of study participants, Saudi Arabia

Bio-demographic data		No	%
Smoking	Yes	220	22.0%
	No	782	78.0%
Chronic diseases	Hypertension	83	8.3%
	Hyperparathyroidism	75	7.5%
	Diabetes mellitus	69	6.9%
	Gout	65	6.5%
	Intestine-related disease	44	4.4%
	Chronic kidney disease	32	3.2%
	None	634	63.3%
Do have/had renal stone before	Yes	161	16.1%
	No	841	83.9%
Family history of renal stones	Yes	420	41.9%
	No	582	58.1%

Prevalence and risk factors of urolithiasis: The study showed that prevalence reached 16.1% of participants. Therefore, participants with a family history of urolithiasis were 1.4 times more likely to develop urolithiasis than those without such a history (OR = 1.38, 95% CI: 1.0-1.9). Among the male participants, 13.2% (73/551) were diagnosed with urolithiasis, while 19.5% (88/451) of the female participants were diagnosed with the condition.

As a result, male respondents had a 15% lower risk of urolithiasis than female respondents (OR = 0.85, 95% CI: 0.58-1.25). The prevalence of kidney stones was likewise substantially lower among Saudis (14.1%) than among non-Saudis (40%). Urolithiasis was reported among 27.7% of participants aged 51 years or more versus 12.3% of those aged 18-25 years. There was a proportional increase in the occurrence of urolithiasis among individuals of higher age as compared to younger individuals (P = 0.002).

Participants with a lower BMI had a higher likelihood of developing renal stones, with urolithiasis diagnosed among 25.5% of respondents who were underweight (OR = 1.9; 95% CI: 1.2-2.3), along with 14.3% of overweight participants, 15.7% of obese participants, and 15.2% of normal-weight participants (Table 3).

**Table 3:** The prevalence of urolithiasis among body mass index categories OR: Unadjusted odds ratio. CI: Confidence interval. * P < 0.05 (significant)

Body Mass Index (BMI)	Prevalence of urolithiasis (%)	OR	95% CI	p-value
Underweight	25.5%	1.9	1.2-3.3	0.017*
Normal weight	15.2%	Reference		
Overweight	14.3%	1.0	0.6-1.4	0.749
Obese	15.7%	1.1	0.7-1.6	0.862

Twenty-two percent of participants were smokers. 30.9% (68/220) of the smokers were diagnosed with urolithiasis versus 11.9% (93/782) of the nonsmokers. Cigarette smokers thus had roughly three times the risk of kidney stone formation (OR = 2.3, 95% CI: 1.6-3.5).

Bivariate analysis was used to determine crude relations. Risk factors were evaluated using a multiple regression method with a backward technique. Four different modules were evaluated, and the factors included in the regression modules were age, gender, diabetes mellitus (DM), body mass index (BMI), cigarette smoking, and family history. The results showed that age, smoking, diabetes mellitus (DM), and body mass index (BMI) were the most significant factors (P < 0.0001). Surprisingly, family history (P = 0.121) was the only factor that was not significant after adjustment (Table 4). 

**Table 4:** Risk factors associated with urolithiasis ORU: Unadjusted odds ratio. CI: Confidence interval. * P < 0.05 (significant)

Factors	p-value	OR_U_	95% CI	
			Lower	Upper
Male gender	0.407	0.85	0.58	1.25
Smoking	0.001*	2.33	1.56	3.47
Family History	0.049*	1.38	1.00	1.99
Diseases	0.001*			
Hypertension (HTN)	0.001*	3.36	1.86	6.07
Diabetes mellitus (DM)	0.001*	4.89	2.68	8.90
Hyperparathyroidism	0.001*	4.90	2.74	8.78
Gout	0.001*	4.07	2.19	7.58
Chronic Kidney disease	0.001*	7.50	3.44	16.32
Intestinal diseases	0.086	2.08	0.90	4.81

## Discussion

Urolithiasis is a disease known for its high prevalence and high recurrence rate. Focusing on this disease is important not only for its treatment but, above all, for its prevention and the identification of its risk factors. This study was therefore carried out to assess the prevalence of urolithiasis among the Bisha population.

The study showed that 16.1% of participants were diagnosed with urolithiasis. This prevalence is higher than reported values in Jeddah and Arar, which were 11.2% and 12.3%, respectively [2,15]. After data analysis, several risk factors for developing urolithiasis were identified, including smoking, family history, and comorbidity. 

The study found male participants were 15% less likely to develop urolithiasis compared with females. On the other hand, other studies showed that males had double the risk of renal stones compared with females [16,17]. An explanation for this gender gap is that females produce large amounts of citric acid due to estrogen, which protects against urolithiasis [4]. That said, recent data suggest that this discrepancy is dwindling due to changes in lifestyle and dietary habits [18].

Furthermore, the prevalence of urolithiasis was higher among those 51 years of age and older. This result is similar to another study conducted by Krambeck et al. [19]. Another study, however, conducted in Jeddah and Riyadh, revealed a high prevalence of urolithiasis (33.70%) among individuals aged 18 to 30 [20]. Regarding the factor of age, our study found that the highest risk of developing urolithiasis was at the end of the patient’s career; this confirms the hypothesis that lifestyle and dietary habits could be risk factors for urolithiasis. 

Not surprisingly, urolithiasis formation was more significant among participants with a family history of urolithiasis; this could be attributed to the type of food consumed by the affected family, as a lot of food staff had a risk for urolithiasis. On the other hand, some of the inherited diseases, like thyroid disease, are known to be risk factors for urolithiasis. A previous study also found a significant association between family history and a two-fold increase in urolithiasis risk [21]. Meanwhile, concerning body mass index (BMI), Taylor et al. found that a high BMI had a significant association with urolithiasis development. The explanation for this was that urinary excretion of calcium oxalate and uric acid increases the risk of forming urolithiasis [22,23]. However, the present study showed that a lower BMI was also associated with a higher risk of renal stone formation, as highlighted by Trinchieri et al. [24].

Moreover, smokers had a three-times greater risk of urolithiasis. One possible reason is that smoking can decrease urinary flow and increase cadmium in the blood, which may induce urolithiasis [25]. Another study also found this [26]. Last, our study also found that diabetic patients had a higher risk of developing urolithiasis. Another study also reported this [27]. Renal handling of ammonium and calcium and the effect of insulin resistance on urine pH have been posited as explanations for the relationship between diabetes and urolithiasis [28].

Limitations

Because this study relied on an online questionnaire, sampling and respondent bias were issues regarding prevalence estimation. The sample size does not reflect the population of the area because it was only a preliminary discovery in a single location, and a more extensive study is therefore recommended for further investigation.

## Conclusions

This study found a high prevalence of urolithiasis among the study population in Bisha, Saudi Arabia. A higher prevalence of the disease was associated with women and increased age. Body mass index (BMI), smoking, and diabetes were identified as the most significant risk factors.

Based on the findings of this study, we recommend adequate public education regarding urolithiasis, its risk factors, and recent prevalence data in the study area, emphasizing the importance of preventing the disease through medical campaigns and social media. More detailed studies must be conducted in the study area, focusing on uncovering information about urolithiasis.
